# Must we press on until a young mother dies? Remifentanil patient controlled analgesia in labour may not be suited as a “poor man’s epidural”

**DOI:** 10.1186/1471-2393-13-139

**Published:** 2013-07-02

**Authors:** Peter Kranke, Thierry Girard, Patricia Lavand’homme, Andrea Melber, Johanna Jokinen, Ralf M Muellenbach, Johannes Wirbelauer, Arnd Hönig

**Affiliations:** 1 Department of Anaesthesia and Critical Care, University Hospitals of Würzburg, Oberdürrbacher Str. 6, Würzburg 97080, Germany; 2 Department of Anaesthesia, University Hospital Basel, Spitalstrasse 21, Basel, CH 4031, Switzerland; 3 Department of Anesthesiology, Cliniques Universitaires Saint Luc, Université Catholique de Louvain, Brussels, Belgium; 4 Department of Anaesthesia, Salem-Spital, Schänzlistrasse 39, 3000, Bern 25, Switzerland; 5 University Children’s Hospital, Josef-Schneider-Strasse 2, Würzburg 97080, Germany; 6 Department of Obstetrics and Gynecology, University Hospitals of Würzburg, Josef-Schneider-Strasse 4, Würzburg 97080, Germany

**Keywords:** Remifentanil, Epidural Analgesia, Labour Pain, Labour Analgesia, Patient Controlled Analgesia, Patient Satisfaction, Healthcare Cost, Healthcare Economics

## Abstract

**Background:**

The epidural route is still considered the gold standard for labour analgesia, although it is not without serious consequences when incorrect placement goes unrecognized, e.g. in case of intravascular, intrathecal and subdural placements. Until now there has not been a viable alternative to epidural analgesia especially in view of the neonatal outcome and the need for respiratory support when long-acting opioids are used via the parenteral route. Pethidine and meptazinol are far from ideal having been described as providing rather sedation than analgesia, affecting the cardiotocograph (CTG), causing fetal acidosis and having active metabolites with prolonged half-lives especially in the neonate. Despite these obvious shortcomings, intramuscular and intravenously administered pethidine and comparable substances are still frequently used in delivery units.

Since the end of the 90ths remifentanil administered in a patient-controlled mode (PCA) had been reported as a useful alternative for labour analgesia in those women who either don’t want, can’t have or don’t need epidural analgesia.

**Discussion:**

In view of the need for conversion to central neuraxial blocks and the analgesic effect remifentanil has been demonstrated to be superior to pethidine. Despite being less effective in terms of the resulting pain scores, clinical studies suggest that the satisfaction with analgesia may be comparable to that obtained with epidural analgesia. Owing to this fact, remifentanil has gained a place in modern labour analgesia in many institutions.

However, the fact that remifentanil may cause harm should not be forgotten when the use of this potent mu-agonist is considered for the use in labouring women. In the setting of one-to-one midwifery care, appropriate monitoring and providing that enough experience exists with this potent opioid and the treatment of potential complications, remifentanil PCA is a useful option in addition to epidural analgesia and other central neuraxial blocks. Already described serious consequences should remind us not refer to remifentanil PCA as a “poor man’s epidural” and to safely administer remifentanil with an appropriate indication.

**Summary:**

Therefore, the authors conclude that economic considerations and potential cost-savings in conjunction with remifentanil PCA may not be appropriate main endpoints when studying this valuable method for labour analgesia.

## Background

Ideal labour analgesic will provide sustained pain relief for the time period when the parturient is in need for analgesia, but is flexible enough to be adjusted to the varying analgesic requirements throughout the labour process and wears off quickly, if analgesia is not needed any more following delivery. Furthermore, the ideal labour analgesia will have a low incidence of side effects for the mother and the baby and will be easy to institute irrespective of the resources and skills available to allow that all parturients who wish to experience significant pain relief actually receive effective analgesics. Moreover, the ideal labour analgesic may depend on the parturient and the individual preferences as well as expectations for the birth experience since maximum pain relief may not always be of utmost importance [1].

According to the guidelines of the American Society of Anesthesiologists (ASA) and the American College of Obstetricians and Gynecologists (ACOG), neuraxial analgesia techniques (epidural, spinal, and combined spinal–epidural) are recommended in labour as “the most flexible, effective, and least depressing to the central nervous system” of the choices available and thus “allowing for an alert participating woman and an alert neonate” [2].

Similar statements and recommendations exist on national levels. The recommendation of the German Society of Anaesthesia and Intensive Care, for instance, states that central neuraxial regional analgesia and anaesthesia are especially safe and effective to provide adequate pain relief in the peripartum period and for anaesthesia during cesarean section without leading to a relevant impairment of the vital signs of the newborn [3].

Owing to the fact that “maternal request is a sufficient medical indication for pain relief during labor”, the threshold to withhold effective pain relief is considerable high. This view is supported by the respective ACOG recommendations stating that: “Labor results in severe pain for many women. There is no other circumstance in which it is considered acceptable for a person to experience untreated severe pain, amenable to safe intervention, while under a physician’s care. In the absence of a medical contraindication, maternal request is a sufficient medical indication for pain relief during labor” [4].

These remarks are well in line with general statements on pain relief and represent the spirit of the Declaration of Montreal of the International Association for the Study of Pain (IASP), stating “that access to pain management is a fundamental human right” [5].

Bearing these essentials in mind – access to pain management as a fundamental human right and maternal request as a sufficient medical indication for pain relief during labour – it is self-evident, that for labouring women who either don’t want, can’t have or don’t (yet) need epidural analgesia, best suited alternative options need to be provided in order to ensure that as much as possible factors, representing the “ideal labour analgesic” will be met.

The following discussion provides a short overview of current analgesic techniques available with special reference to intravenous remifentanil, administered in a patient controlled manner. Further, suggestions for future topics of research with the intention to provide more solid data when and how its use should be recommended to ensure safety for mother and baby as an utmost clinical and medicolegal goal will be given. In addition, the discussion should raise awareness that the anesthesiologist in the delivery suite should not solely be regarded as a prerequisite to ensure labour analgesia via the epidural route but is equally important when it comes to risk assessment of the labouring woman and to ensure that emergency situations (including emergency cesarean section) can be adequately managed.

Last but not least the article should stimulate instructive discussions, whether cost-minimization represents a suitable approach to assess the suitability of alternatives to central neuraxial labour analgesia, as outlined and emphasized in a recently published study protocol in this journal [6].

## Discussion

### Long-acting parenterally administered opioid analgesics

Many parenterally administered opioid analgesics have been tested so far in the aim to provide alternatives for central neuraxial analgesia in the parturient, either for those parturients who do not wish to receive central neuraxial analgesia or in settings where methods of central neuraxial analgesia are not available or medically contraindicated.

The advantage of systemic analgesia may be that it’s use is not restricted to settings where health professionals being qualified to perform central neuraxial blocks are available and appropriate monitoring and the ability to cope with complications cannot be ensured. Unfortunately, despite extensive search for alternative options and long-lasting experience with various options, real competitive options to central neuraxial analgesia have not been elucidated so far.

Unlike in the postoperative or acute trauma setting, where pethidine has not a place any more [7], pethidine and meptazinol – and to a lesser degree morphine – still remain popular and widely used options for pain relief during labour. In view of the overall limited effect and well-recognized side-effects [8-10] the persisting dominance of these opioid analgesics remains striking. In a Norwegian survey published in 2009 pethidine was the most commonly used opioid analgesic, being administered in a nurse controlled fashion in 77% of the units who responded to the survey, while the epidural frequency was only 26% [11]. The survey further found out that “Nurse-administrated analgesia was the method of choice for all units, either i.v. or intramuscular (i.m.)”. It was striking that in this survey fifty-eight per cent reported only i.m. administration, 8% i.v. and 34% both forms. A survey conducted in Germany only recently suggested that parenteral opioids remain a popular offer for women with labour pain [12]. Despite the fact that all departments responding to the survey provided a 24 h epidural service, “the most commonly used opioids were pethidine (19%), meptazinol (17%) and piritramide (16%) for intermittent intravenous/intramuscular administration” [12]. In case of the use of a PCA device to administer the analgesic, remifentanil was already the most popular choice (68%).

It remains not only the choice of the drugs itself, but especially the route of administration that lacks scientific evidence and is considered outdated, e.g. in the postoperative period, to cope with acute pain [7]. What is already common practice in the postoperative care has not reached the delivery suites yet, with intramuscular route still being a popular route of administration. Although the evidence with any of these drugs given intravenously is much poorer than with the intramuscular route it remains unchallenged that “The intramuscular route is not recommended because it is not dependable – the rate of drug-absorption may vary. Intravenous administration is more reliable,….” These recommendations also apply to so-called “low-resource settings”, as the Chapter 17 on “Pharmacological Management of Pain in Obstetrics” of the IASP Guide to Pain Management in Low-Resource Settings points out [13].

The current evidence regarding parenteral opioids for labour pain is comprehensively summarized in a recent Cochrane Review that concluded “Parenteral opioids provide some relief from pain in labour but are associated with adverse effects. Maternal satisfaction with opioid analgesia was largely unreported but appeared moderate at best” [10].

More accentuated views are presented in the original trials that often suggest “that labour pain is not sensitive to systemically administered morphine or pethidine” and that “These drugs only cause heavy sedation. It therefore seems unethical and medically incorrect to meet parturients' requests for pain relief by giving them sedation. Considering the well documented negative effects on newborn infants we also believe systemic pethidine should be avoided in labour” [8]. Single trials also report a rather short-lived effect of traditional opioids when they draw the conclusion that “Every woman receiving one injection of meptazinol complained of moderate to severe pain after 2 h; 97% of those receiving one injection of pethidine were complaining of moderate to severe pain after 2 h” [14].

And in a comparison with meptazinol, once introduced with the expectation to cause lower incidences of opioid adverse effects, Nel et al. who compared meptazinol and pethidine in a double-blind randomized trial with regard to analgesia during the first stage of labour as early as 1981 stated “… that neither drug is effective for sustained pain relief, and that there is no advantage of one over the other” and further remarked that “The critical reassessment of traditional drugs for analgesia in labour is suggested” [15].

### Inhaled nitrous oxide

In view of the apparent need to have choice in pain relief and the frequently observed tendency to first opt for less invasive methods of pain relief, the popularity of inhaled analgesia is not surprising. The popularity of nitrous oxide varies from country to country. While it is used by about 60% of laboring women in the UK and is also frequently used in Australia and the Scandinavian countries [11-16], it is rarely available as an analgesic option in Germany or the USA.

In principle, these methods ensure that the mother stays awake and laryngeal reflexes remain intact. The fact that inhaled interventions for pain relief are usually easy to administer with limited preparation time and fast onset account for their popularity in some countries.

Recently, the existing body of evidence with respect to nitrous oxide and other inhaled molecules have been subjected to a systematic review in which the authors concluded that “Inhaled analgesia appears to be effective in reducing pain intensity and in giving pain relief in labour” [17]. However, these conclusions have been based mainly on the fact that placebo, sham-treatment or no treatment provided less pain relief compared to nitrous oxide (average RR 0.06, 95% CI 0.01 to 0.34, two studies, 310 women; MD −3.50, 95% CI −3.75 to −3.25, one study, 509 women). Based on the report of 8 controlled trials and in 8 observational studies another systematic review had summarized the findings of these trials in a systematic review slightly different, stating that “Nitrous oxide is not a potent labor analgesic, but it is safe for parturient women….” [18].

It goes without saying that even this ‘innocent’, because less invasive, intervention is associated with more side effects for women such as nausea (RR 43.10, 95% CI 2.63 to 706.74, one study, 509 women), vomiting (RR 9.05, 95% CI 1.18 to 69.32, two studies, 619 women), dizziness (RR 113.98, 95% CI 7.09 to 1833.69, one study, 509 women) and drowsiness (RR 77.59, 95% CI 4.80 to 1254.96, one study, 509 women) when compared with placebo or no treatment [17]. These findings support the notion, that it cannot be argued that such an intervention should be tried first, with the argument that no relevant adverse effects will occur. In addition it might be important to note that nitrous oxide is a greenhouse gas and reacts with ozone. More recently there is increasing evidence of neuroapoptosis induced by anesthetic agents in the developing brain. The role of nitrous oxide in this context is unclear [19].

In a study hypothesizing that remifentanil would provide better analgesia during labour than nitrous oxide, the authors compared intravenous remifentanil in 0.4 μg/kg PCA doses with 1-min infusion and lock-out times with intermittent inhaled 50% nitrous oxide during a 20-min study period with a subsequent 20-min wash-out sequence after each period [20]. The main result had been that the “median decrease in pain score for remifentanil was 1.5 and that for nitrous oxide 0.5 (P=0.01)” on a verbal numeric rating scale from 0 to 10. Furthermore, “the parturients gave better pain relief scores with remifentanil than with nitrous oxide” with a median of 2.5 (remifentanil) vs. 0.5 (nitrous oxide), respectively. In contrast to these more beneficial effects “sedation was reported more often, and SaO_2_ was slightly lower during remifentanil administration” Although blinding was ensured, most patients preferred the analgesia provided by remifentanil to that provided by nitrous oxide (14 vs 1, out of 15 parturients who completed the study).

### Remifentanil

Remifentanil is available on the market in Europe since the 90’s. In Germany, being available since 1996, it is licensed to supplement general anaesthesia as a μ opioid agonist or for the use as analgesic in conjunction with sedation and analgesia on ICU. Although not very popular in clinical care, its use as analgesic in the postoperative period for the awake and spontaneous breathing patient is mentioned in the Summary of Product Characteristics (SPC). In this regard a continuous infusion (start with 0,1 μg/kg/min) is recommended in the SPC, while the administration of boluses in the spontaneous breathing patient is “not recommended” according to the wording of the SPC.

Owing to its unique pharmacokinetic properties with rapid metabolism by tissue esterase, its use has been quickly widened out to the use for labour analgesia, since for the first time in the history of opioid analgesia provided by the parenteral route “adequate doses of opioid (could be) administered to the mother to achieve good analgesia, without its accumulation in the fetus” [21,22] the use of remifentanil has been extended from patients presenting with (relative) medical contraindications for neuraxial blockade to patients who refuse epidural analgesia (e.g. due to backache) [23] or to settings, where neuraxial analgesia is simply not available [24].

Concomitantly, the use of remifentanil gained popularity for the supplementation of a general anaesthesia in the cardiovascular compromised obstetric patient, e.g. aortic or valvular disease [25] or patients presenting with a cardiomyopathy [26].

Until now, the available published evidence suggests that even the administration of remifentanil immediately before cord clamping (e.g. at induction of general anaesthesia for cesarean delivery) does not mean that the neonate is at significantly increased risk to need mask ventilation or tracheal intubation [27] although the studies looking at this outcome are limited and the event rate is still very low, limiting the conclusions that can be drawn. Furthermore, and owing to own experiences, these endpoints are highly variable with the experience and knowledge of the neonatal team caring for the newborn in that regard, that neonatologists knowing the substance and its unique pharmacokinetic profile tend to be more conservative regarding early airway interventions.

These encouraging data lead to the recommendation of national societies to supplement general anaesthesia with an adjunct to attenuate the cardiovascular response to intubation and skin incision in the parturient with preeclampsia [3].

The use of remifentanil, though not uniformly supported by all available evidence, gained more and more popularity and is in routine use in some institutions, not only in cases where central neuraxial blocks are contraindicated [28,29].

The cumulative evidence suggests that compared with pethidine, its use for labour analgesia is associated with significantly improved pain relief and satisfaction and is associated with a significantly decreased conversion rate to epidural analgesia [30]. Trials comparing intravenous patient controlled analgesia using remifentanil with epidural analgesia suggest – which is not a surprise – a more pronounced reduction in pain scores with neuraxial analgesia [30,31]. When it comes to overall satisfaction with the analgesic technique, however, the studies report that “Maternal satisfaction was similar” [31] or “Patient satisfaction scores during and after delivery were similar in both groups” [32].

In view of the neonatal outcome, the available evidence does not suggest a negative effect on the newborn. This is not surprising, considering the results obtained in clinical trials where remifentanil had been administered immediately prior to cord clamping in conjunction with general anaesthesia for caesarean section [27].

Clearly, the potential to cause adverse effects as a potent μ-agonist must not be forgotten [33,34] and subsequently has raised concerns in view of its use for labour analgesia and evoked the provocative question used for this article: “Must we press on until a young mother dies?” [35]. The fact that labour analgesia is not a licensed indication may have further prevented its use in many institutions so far [36]. Although it has to be emphasized – from a medicolegal point of view – that the lack of an indication (as stated in the SPC) is not a sufficient justification to withhold treatment. On the contrary! Knowing that alternative options – may be even safer or effective – do exist, would necessitate to inform patients accordingly and use such an intervention irrespective of its indication as stated by the manufacturer in the SPC [37-40].

### Remifentanil – what we would like to know

Although in the meanwhile new evidence suggests that remifentanil will have a role to cope with labour pain [41], any new evidence to support its use and elucidate pitfalls and shortcomings may be welcome in order to promote a safe use and not harm the mother or neonate. This is especially true since, at least so far, there seems to be little agreement regarding the necessary monitoring during its use in the labour suite [12]. The latter is true also for other opioids, since these are associated with decreases in oxygen saturation, sedation and the need for oxygen administration as well [42].

Persistent pain following labour is an important socioeconomic factor and has been found to be as frequent as 1 in 10 women [43]. On the other hand remifentanil has shown to possess a potential to cause opioid-induced hyperalgesia [44]. Therefore, the role of remifentanil PCIA in persistent and chronic pain following childbirth should be addressed in subsequent evaluations.

There is an ongoing discussion on the suitability of various regimens to administer remifentanil as labour analgesic. While some argue that a continuous background infusion may be helpful, others argue – in accordance with recommendations to ensure safety of opioid patient controlled analgesia in the postoperative setting [45] – that the avoidance of any background infusion may constitute an essential safety feature of this highly potent μ-opioid in that respect that any potential respiratory depression associated with a self-administered bolus should be short-lived.

In view of these recent developments, we were highly pleased to read the study protocol describing a randomised controlled trial investigating remifentanil patient controlled analgesia versus epidural analgesia during labour (RAVEL protocol) [6].

In a multicentre randomized controlled study performed at various Dutch hospitals the objective of the study “is to test the hypothesis that remifentanil PCA is as effective as epidural analgesia with respect to patient satisfaction and pain appreciation scores with lower costs and possibly benefit of easier achievement of 24 hours availability of pain relief for women in labour” [6].

We would like to challenge the assumption that overall costs and ease of administration are meaningful and sensible endpoints in this setting.

There is no doubt, that in principle and taking into account all costs of an epidural service and providing a stand-by service with an anesthesia team in general (contingency costs) costs more than simply providing a patient suffering from labour pain and requesting for pain relief with an intravenous PCIA-pump (patient controlled infusion pump). However, this assumption neglects the statement which is valid so far, that remifentanil PCIA should not be viewed as an full substitute for epidural analgesia and in many institutions that extensively practice this method of pain relief is viewed as complementary tool either for those cases not needing an epidural catheter or other forms of neuraxial pain relief or before changing to neuraxial analgesia [23,28,40].

Although it may be acknowledged that in low resource settings, the availability of remifentanil could attenuate suffering in conjunction with childbirth experience, this should not be the leading argument in developed countries, where standards have been established and birth rates are declining. In these settings, the primary aim should be to hold up the established standards in view of safety and to this aim, anaesthesiologists may contribute a lot more than simply inserting epidurals [46].

It goes without saying that the indication to prescribe remifentanil, at least when administered in analgesic doses necessary to obtain pain relief during labour, should stay in the hands of personnel having experiences with the substance and being able to cope with potential side effects.

Lastly, the obtained outcomes should be subject to a close quality control program to gain future insights and ensure that remifentanil retains its valuable place in the delivery suite.

To our knowledge there is only one published study, in which remifentanil was administered as a continuous infusion in a setting where no anaesthetist has been available. In view of the potential to cause harm [33], we would also challenge the general assumption, “that the presence of an anesthetist for this type of analgesia is not required and that administration of remifentanil is quicker and less invasive than epidural analgesia” [6]. At least, this assumption contradicts the SPC in Germany, where high hurdles are stated in view of the personal that should be allowed to administer this potent opioid. We would rather agree with Tveit and colleagues stating that “Remifentanil intravenous patient-controlled analgesia provides adequate pain relief and high maternal satisfaction during the first and second stages of labour”. but also reminded us that “Maternal sedation and respiratory depression may occur,…” and therefore “careful monitoring is mandatory” [47].

Owing to the fact that cost analyses are highly variable – consider for instance the performance of a MRI-Scan to exclude epidural abscess or hematoma following epidural analgesia – we would assume that the outcome in terms of costs may yield any desired values depending on the models applied.

To our understanding the aim to assess the possible “benefit of easier achievement of 24 hours availability of pain relief for women in labour” [6] cannot be answered since an epidural service needs to be hold available anyway due to the performed randomization to either the epidural or the remifentanil PCIA group. Performing “epidural analgesia according to local protocol” [6] is a pragmatic design and per se not criticisable. However, considering the fact that “epidural analgesia” may imply quite different protocols, we suspect that this may render the comparison versus remifentanil highly variable with regard to the outcome “pain appreciation”.

The authors all use this form a labour analgesia and are convinced that it fits well into the armamentarium of labour analgesia available so far. More importantly, it constitutes an alternative for women who either don’t want, can’t have or don’t (yet) need epidural analgesia or want to try an alternative option to central neuraxial analgesia first. Therefore, clinical trials and observational data assessing the risk for maternal fever, assessing the beastfeeding success, drop in oxygen saturation, satisfaction, APGAR values, Neurologic and Adaptive Capacity Score (NACS) amongst other well-established outcomes are welcome.

However, the authors are concerned that if the limitations of remifentanil PCA as a potent intervention are not strictly taken into account and economic reasoning is overbearing, we may put too much pressure on this valuable intervention to succeed and thus “press on until a young mother dies” [35].

John Snow, by royal invitation, used chloroform on Queen Victoria during the delivery of Prince Leopold on 7th April 1853. At that time, the medical establishment, in view of the reported side effects, and with an innate resistance to clinical advances, commented in the Lancet [48]: “Intense astonishment, therefore, has been excited throughout the profession by the rumour that her Majesty during her last labour was placed under the influence of chloroform, an agent which has unquestionably caused instantaneous death in a considerable number of cases. Doubts on this subject cannot exist”.

These caveats are nicely illustrated by anecdotal evidence about a Mr. Gladstone, who was Prime Minister at the time of Prince Leopold’s birth. When he visited the sovereign to discuss affairs of state Gladstone congratulated the Queen on the addition to her family, and then asked her how she liked Dr Snow’s chloroform. The Queen replied ‘Very well, Mr Gladstone’. He replied ‘The bishops are not pleased’. ‘Then let the bishops have the babies Mr. Gladstone’ she replied.

At these times, nobody would have imagined that labour analgesia would be as self-evident in conjunction with childbirth as it is in many institutions around the world today. And remifentanil may be one day as self-evident in the labour suite as in the perioperative setting or as pain relief itself during labour and the regimen may further vary depending on the setting in which it is used [49]. However, anaesthesiologists should be cautious and concerned with ensuring that such a valuable method is not discredited with complications occurring due to the fact that safety standards are not guaranteed or cost issues dominate the discussion. We should bear in mind, that remifentanil patient controlled analgesia in labour so far should not be viewed as a poor man’s epidural requiring less monitoring as compared to epidural analgesia [50].

## Summary

Multiple beneficial effects when compared with conventional opioids contributed to the fact that remifentanil PCA has already gained a place in modern labour analgesia in many institutions.

However, remifentanil retains its potential to cause harm also in the labouring women. In the setting of appropriate precautions, such as one-to-one midwifery care, appropriate monitoring and providing that enough experience exist with this potent opioid including its side effects, remifentanil PCA is a useful option in addition to epidural analgesia and other central neuraxial blocks, provided that adequate safety precautions are met. Thus, for various reasons remifentanil should be viewed as complimentary method to the more potent neuraxial techniques and not as a “poor man’s epidural”. Cost-savings may only be realized by reducing vital precautions and thus leading to catastrophic outcomes, with the risk to cast a damning light on this valuable armamentarium for labour analgesia.

Therefore, the authors conclude that economic considerations and potential cost-savings in conjunction with remifentanil PCA may not be appropriate endpoints when studying this valuable method for labour analgesia [51].

## Competing interests

AM maintains a quality assurance project for the use of remifentanil PCA as labor analgesic http://www.remipca.org and provides services for institutions aiming to adopt this method of labor analgesia. PK gives lectures and provides implementation help in conjunction with remifentanil PCA as labor analgesic (academic basis, not for profit).

## Authors’ contributions

All authors made substantial contributions to the conception of the article. PK wrote a first draft and coordinated the feedback from the co-authors to provide subsequent versions of the article. All authors read and approved the final manuscript.

## Authors’ information

PK is Consultant anaesthetist and Professor of Anaesthesia at the Department of Anaesthesia and Critical Care. He is responsible for the anaesthesia services in obstetrics and gynecology at Würzburg University Hospital. TG is Consultant anaesthetist and Professor of Anaesthesia. He is responsible for the anaesthesia services in obstetrics and gynecology at Basel University Hospital. PL is Consultant anaesthetist and Professor of Anaesthesia at the Department of Anesthesiology, Cliniques Universitaires Saint Luc, Université Catholique de Louvain, Brussels, Belgium, AM is Consultant anaesthetist at the Department of Anaesthesia, Salem-Spital, Bern, Switzerland, JJ is resident in the Department of Anaesthesia at Würzburg University Hospital, RMM is Consultant anaesthetist. He is responsible for the Critical Care Unit of the Department of Anaesthesia at Würzburg University Hospital. JW is Chief Consultant Neonatologist at University Children’s Hospital, Würzburg, Germany. AH is Deputy Director of the Department of Obstetrics and Gynecology at the University Hospital of Würzburg.

## Pre-publication history

The pre-publication history for this paper can be accessed here:

http://www.biomedcentral.com/1471-2393/13/139/prepub
